# The changing biodiversity of Alabama *Drosophila*: important impacts of seasonal variation, urbanization, and invasive species

**DOI:** 10.1002/ece3.2452

**Published:** 2016-09-12

**Authors:** Andrei Bombin, Laura K. Reed

**Affiliations:** ^1^ Department of Biological Sciences University of Alabama Tuscaloosa AL USA

**Keywords:** biodiversity, *Drosophila*, invasive species, seasonal variation, subtropical climate, urbanization

## Abstract

Global warming and anthropogenic disturbances significantly influence the biosphere, tremendously increasing species extinction rates. In Central Alabama, we analyzed Drosophilidae species composition change nearly 100 years after the previous survey. We found ten Drosophilid species that were not reported during the last major biodiversity studies, two of which are invasive pests. In addition, we analyzed the influence of seasonal environmental variables characteristic of the subtropical climate zone on *Drosophila* abundance and biodiversity. We found a significant correlation between temperature and abundance of total *Drosophila* as well as for six of the seven most represented species individually, with a maximum abundance at intermediate temperatures (18–26°C). In addition, temperature was positively correlated with biodiversity of *Drosophila*. Precipitation produced a significant effect on the abundance of five species of Drosophila, with different optima for each species, but did not affect overall biodiversity. We concluded that in the subtropical climate zone of Central Alabama, seasonal temperature and precipitation changes produce a significant effect on *Drosophila* abundance and biodiversity, while local land use also impacts fly abundance, contributing to an apparent shift in species composition over the last century. We expect global climate change and other anthropogenic factors to further impact *Drosophila* species composition in the subtropical climate zone into the future.

## 1 Introduction

In the face of global warming, we can see the influence of changes in the world's climate on the biosphere. Currently, the global species extinction rate is 100–10,000 times higher than a predicted natural rate, which suggests that a new mass extinction is underway (Ceballos et al., 2015; Pimm, Russell, Gittleman, & Brooks, 1995). A decrease in global biodiversity may cause serious consequences for the biosphere by influencing ecosystem function and reducing overall productivity of biological communities (Cardinale et al., 2006; Gamfeldt, Hillebrand, & Jonsson, 2008; Hector et al., 1999; Tilman, Wedin, & Knops, 1996). Decreases in ecosystem productivity can lead to serious implications for fishery, forestry, and other types of industries that depend on harvesting natural resources. In addition, many wild species of plants and animals potentially harbor undiscovered beneficial natural products; thus, their extinction could have negative effect on native human populations (Cooper, 2004; Newmark, 2002). Native species can also prevent or slow invasive pests’ habitat range expansion, via interspecific competition (Commar, da Conceição Galego, Ceron, & Aparecida Carareto, 2012; Hooper et al., 2005). Consequently, biodiversity surveys may reveal early colonization of pest species and native species extinction, which makes them a useful tool for natural resource management and prevention of natural habitat degradation.

The Drosophilidae family consists of about 4,000 species distributed in approximately 65 genera, including *Drosophila* (Brake & Bächli, 2008; da Mata, Valadão, & Tidon, 2015; Srinath & Shivanna, 2014). Members of *Drosophila* genus are widely used in genetic, developmental, and molecular biology studies (e.g., Guruprasad, Hegde, & Krishna, 2010; da Mata et al., 2015; Strickberger, 1962). However, relatively little is known about the ecology of *Drosophila* (da Mata et al., 2015; Shorrocks, 1975). Phylogenetic relationships of the *Drosophila* members are not fully understood, and presently *Drosophila* is a paraphyletic group (Robe, Valente, Budnik, & Loreto, 2005). However, several molecular and morphological studies accept that *Drosophila*,* Sophophora*,* Hirtodrosophila*,* Mycodrosophila*,* Zaprionus*, and *Scaptomyza* collectively form one monophyletic clade (Da Lage et al., 2007; van der Linde & Houle, 2008; van der Linde, Houle, Spicer, & Steppan, 2010; O'Grady & DeSalle, 2008; Remsen & O'Grady, 2002).

*Drosophila* has the potential to become a standard model organism for investigating the influence of climate and habitat change on the biological community, as most species have rather limited realized niches and are dependent on a particular group of host organisms for feeding and reproduction (Parsons, 1991; Poppe, Valente, & Schmitz, 2013). In addition, *Drosophila* are sensitive to shifts in essential climate variables (Parsons, 1991; Poppe, Valente, & Schmitz, 2013; Srinath & Shivanna, 2014). The influence of seasonal changes on *Drosophila* abundance and biodiversity has been surveyed mostly in tropical and temperate climatic zones (Dobzhansky & Pavan, 1950; Levitan, 1954; da Mata et al., 2015; Patterson, 1943; Shorrocks, 1975). The abundance of *Drosophila* species is influenced primarily by precipitation in tropical regions (Dobzhansky & Pavan, 1950; Guruprasad et al., 2010; Srinath & Shivanna, 2014; Torres & Madi‐Ravazzi, 2006), while in temperate climate regions, seasonal changes in abundance and biodiversity of *Drosophila* are better explained by temperature variation (da Mata et al., 2015; Patterson, 1943; Poppe, Valente, & Schmitz, 2013).

In addition, *Drosophila* has the potential to be a model organism for studying the influence of anthropogenic disturbance, particularly urbanization, on species biodiversity and abundance. Endemic *Drosophila* species are often very sensitive to natural habitat deterioration, can form very specific host associations, and are not able to adapt to human environmental modifications (Avondet, Blair, Berg, & Ebbert, 2003; Dobzhansky & Pavan, 1950; Ferreira & Tidon, 2005; van Klinken & Walter, 2001; Parsons, 1991; Shorrocks, 1975). As a result, a decrease in abundance of endemic species of *Drosophila* could indicate that a habitat is recently or currently being disturbed by human influence. The opposite is true for cosmopolitan species of *Drosophila* that are often abundant in urban environments (Avondet, Blair, Berg, & Ebbert, 2003). During a biodiversity survey in an urban area in Brazil, it was shown that exotic species of *Drosophila* contributed to over 90% of the total abundance in this type of environment (Ferreira & Tidon, 2005).

Surveys of wild populations of cosmopolitan *Drosophila* are especially important as there are two known pest Drosophilids*: Drosophila suzukii* and *Zaprionus indianus*. Both of these pest species are polyphagous and are very efficient in colonizing new types of environments (Burrack, Smith, Pfeiffer, Koeher, & Laforest, 2012; Commar et al., 2012). *Drosophila suzukii* originated in Asia and has recently quickly expanded its range, being first reported in the continental United States in 2008 and in Europe in 2009 (Berry, Anthony, Newfield, Ornsby, & Armstrong, 2012). In new habitats, *D. suzukii* broadened its host range and became a significant pest of berries and soft flesh fruits such as plums, peaches, and nectarines (Berry et al., 2012; Burrack et al., 2012). *Zaprionus indianus* is believed to have originated in Africa where it was not considered a significant pest (Commar et al., 2012; Joshi, Biddinger, Demchak, & Deppen, 2014). However, in 1999, *Z. indianus* was reported in South America, where it became a serious pest of figs (Commar et al., 2012; Vilela, 1999). In 2005, *Z. indianus* was reported in North America, Florida, where it continued to expand its habitat and host range (Commar et al., 2012; van der Linde et al., 2006).

To the best of our knowledge, there is very little accessible information on the influence of seasonal climate variables on abundance of *Drosophila* in subtropical regions, specifically Alabama. The average annual temperature for Central Alabama is 18.3°C and varies monthly from 1.6 to 33.7°C, with average annual precipitations of 1395 mm, ranging from 90 to 142 mm per month (NOAA 2014–2015). The last major *Drosophila* biodiversity survey reported the presence of 26 Drosophilidae species in Alabama (Sturtevant, 1916, 1918, 1921). However, the influence of climatic variables on abundance and biodiversity was not evaluated. Due to the relatively high seasonal variation in temperature and precipitation in Alabama, we hypothesized that both of these climatic variables will significantly influence *Drosophila* abundance and biodiversity. In addition, we hypothesized that abundance of cosmopolitan *Drosophila* species would be higher in urban settings than in industrial or minimally disturbed rural environments.

## 2 Materials and Methods

### 2.1 Sample collection

**Figure 1:**
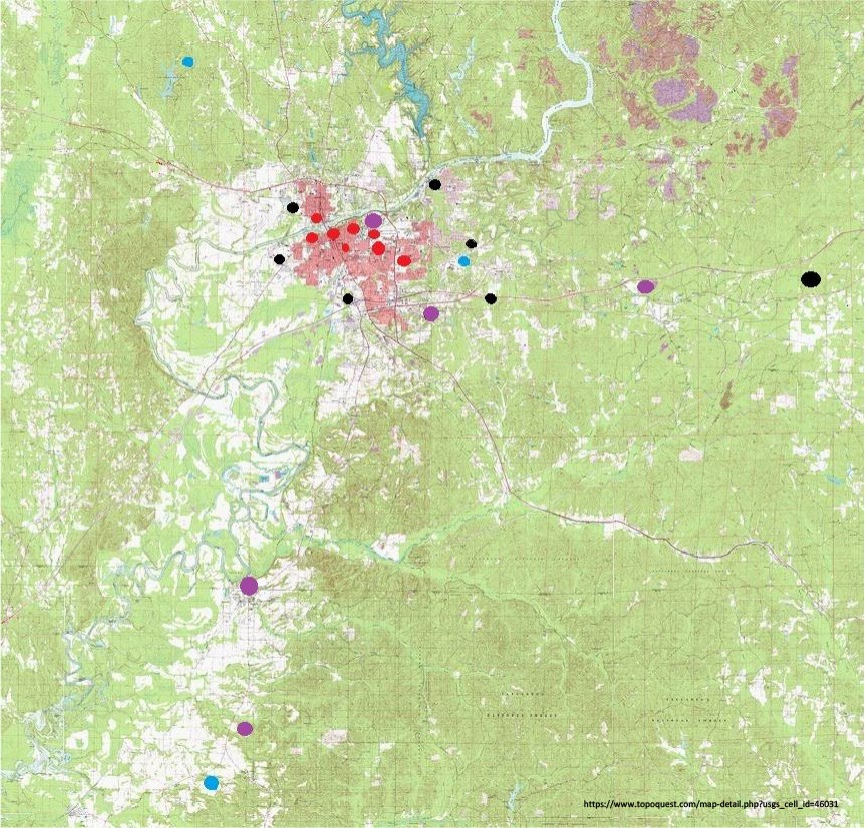
Collection sites around Tuscaloosa. Nonurban parks are marked in blue, urban parks are marked in red, sites of industrial production and industrial product storage are marked in black, and sites that do not match any of the described categories are marked in purple

We collected samples from 23 sites in and around Tuscaloosa, AL (Fig. 1 and Table S1). Collection sites were chosen based on land‐use type. We sampled seven sites that are used for industrial production or storage of industrial products, eight urban parks, three nonurban parks (a biological station, an arboretum, and a state park), and five sites that did not fall into any category (a highway rest station, an apartment complex, an archeological park, a roadside, and a farm, Table S1). Latitude and longitude of each collection site were recorded with a Garmin GPS navigator (Table S1). Samples were collected from banana and mushroom traps left overnight and collected in a time range from 7:00 a.m. to 11:00 a.m. the following morning (18–24 hr collection period total) using an alcohol aspirator (Markow & O'Grady, 2005). Samples were stored in 70% ethanol at −20°C. We made 16 collection trips from July 2014 to May 2015. Collection trips were performed once per month except for the time period from August 2014 to November 2014, during which collections were performed twice per month. For the period from June 2014 to December 2014, we sampled from five to seven randomly selected sites from our 23 collection sites, then starting in January 2015, we chose six collection sites to focus on and visited them monthly.

### 2.2 Sample identification

Samples were identified based on species identification keys (Markow & O'Grady, 2005; Strickberger, 1962). DNA from representative samples of each species was extracted via DNA extraction Chelex 100 protocol (Walsh, Metzger, & Higuchi, 1991), modified by usage of one *Drosophila* sample per DNA extraction. Extracted DNA was PCR‐amplified for the cytochrome oxidase 1 (CO1) gene (Reed, Nyboer, & Markow, 2007) and then sent for sequencing. Morphological species identifications were confirmed with CO1 sequence similarity to Genbank reference sequences via BLAST.

### 2.3 Seasonal climatic data

Climate data were obtained from the Tuscaloosa weather station of the National Oceanic and Atmospheric Administration (NOAA). Average temperature was calculated via averaging daily high and low temperatures. Monthly averages were calculated via averaging daily temperature and precipitation for the 2‐day collection period and the preceding 28 days. In addition, we calculated the average climate variables for each month that preceded each collection month (56–28 days preceding).

### 2.4 Statistical analyses

We limited our analyses to collected representatives of *Drosophila*,* Sophophora*,* Hirtodrosophila*,* Mycodrosophila*,* Zaprionus*, and *Scaptomyza*. According to several studies, these groups form a monophyletic group: *Drosophila* Genus Complex (van der Linde et al., 2010; O'Grady & DeSalle, 2008; van der Linde & Houle, 2008; Da Lage et al., 2007; Remsen & O'Grady, 2002; FlyBase). All statistical analyses were performed using BiodiversityR package, version 2.5–4 according to the BiodiversityR manual (Kindt & Coe, 2005). Influence of climatic variables on the abundance of all *Drosophila*,* Mycodrosophila*, and *Scaptomyza* samples, as well as on each species individually was performed with negative binomial GLM of the following form:

g(log(abundance))=a+b×climate variable+deviation

In our work, we refer to abundance as a number of individual specimens that were collected during a single collection trip at a given site. The influence of each climatic variable on species abundance was tested with linear and quadratic models. The final single‐term model was chosen based on the lowest Akaike information criterion (AIC) value. In addition, with linear and quadratic negative binomial GLM models, we tested the influence of the precipitation average in month previous to the collection month on the abundance of mycophagous *Drosophila* species: *D. tripunctata* and *D. putrida*. We also tested linear and quadratic multivariate models that combined effects of temperature and precipitation. The best multivariate model was chosen based on AIC value and single‐term deletion test. The multivariate model was considered superior if it explained more deviance and has a lower AIC value than models with only one variable. Biodiversity was evaluated as species richness and the Shannon and Simpson biodiversity indices. To analyze the influence of climate variables on biodiversity, we used linear regression models of the following form:

y=a+b×climate variable+deviation.

To evaluate the influence of land use on abundance and biodiversity of *Drosophila,* we separated 17 of our sites into three land‐use categories: nonurban parks, urban parks, and places of industrial production. To evaluate sufficiency of the number of sampling sites for each category, rarefaction analyses were performed (Kindt & Coe, 2005). In addition, we developed a model that evaluated all of our sites for the presence or absence of six variables: garbage or trash cans, industrial or agricultural production, asphalt road, highway, railroad or airport, and residential or public catering buildings. The site's degree of anthropogenic disturbance (disturbance score) was the sum of factors present for each collection area and was then tested in a negative binomial GLM against *Drosophila* abundance per trap. Linear regression was used to test for correlations between biodiversity (as measured by species richness, Shannon and Simpson indices) and the disturbance score. In addition, using the negative binomial model, we tested the influence of latitude and longitude on the abundance and biodiversity of *Drosophila*.

## 3 Results

### 3.1 Collected species

**Figure 2:**
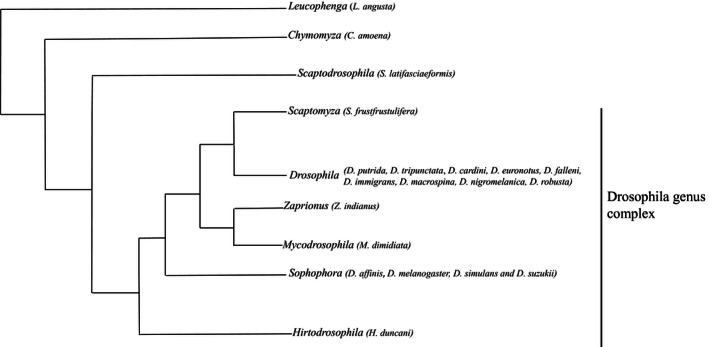
Simplified phylogenetic tree of Drosophilidae. Built on the basis of O'Grady and DeSalle (2008), and Remsen and O'Grady (2002) studies. Drosophila species that we collected in each Drosophilidae's group are shown in parentheses

**Figure 3:**
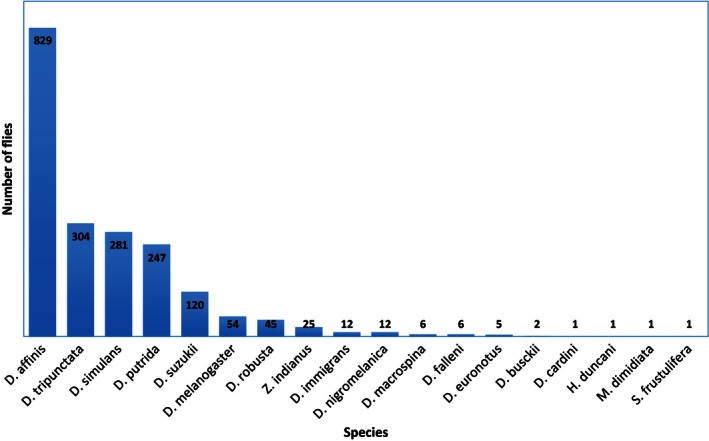
Number of individual flies per species collected from June 2014 to May 2015 and used for statistical analysis

During our collections, we found 21 Drosophilidae species. We collected and identified 14 species of named *Drosophila* genus: *D. affinis*,* D. putrida*,* D. tripunctata*,* D. melanogaster*,* D. simulans*,* D. suzukii*,* D. busckii*,* D. cardini*,* D. euronotus*,* D. falleni*,* D. immigrans*,* D. macrospina*,* D. nigromelanica*,* D. robusta*, and four closely related *Drosophilidae* species: *Hirtodrosophila duncani*,* Mycodrosophila dimidata*,* Zaprionus indianus*, and *Scaptomyza frustfrustulifera* (Fig. 2). Other Drosophilidae species that were identified but not included in statistical analyses were *Scaptodrosophila latifasciaeformis*,* Chymomyza amoena*, and *Leucophenga angusta* (Fig. 2), because these species fell out of the monophyletic *Drosophila* Genus Complex*. D. affinis*,* D. putrida*,* D. tripunctata*,* D. melanogaster*,* D. simulans*,* D. suzukii*, and *D. robusta* were responsible for 96.3% of the total abundance among our samples*. Drosophila affinis* was the most abundant species, contributing to over 42% of total *Drosophila* abundance (Fig. 3).

### 3.2 Climatic variable influence on abundance

We analyzed the influence of climate variables on total abundance of *Drosophila* and closely related species, as well as on the abundance of the most represented species individually. It should be noted that in this manuscript, observed abundances of flies are the result of the combined effects of their actual numbers present in the environment, but also of their activity level and thus “catchability.” When conditions are less favorable over the short term (e.g., too cold), many species of flies become much more difficult to trap.

### 3.3 Overall fly abundance

**Figure 4:**
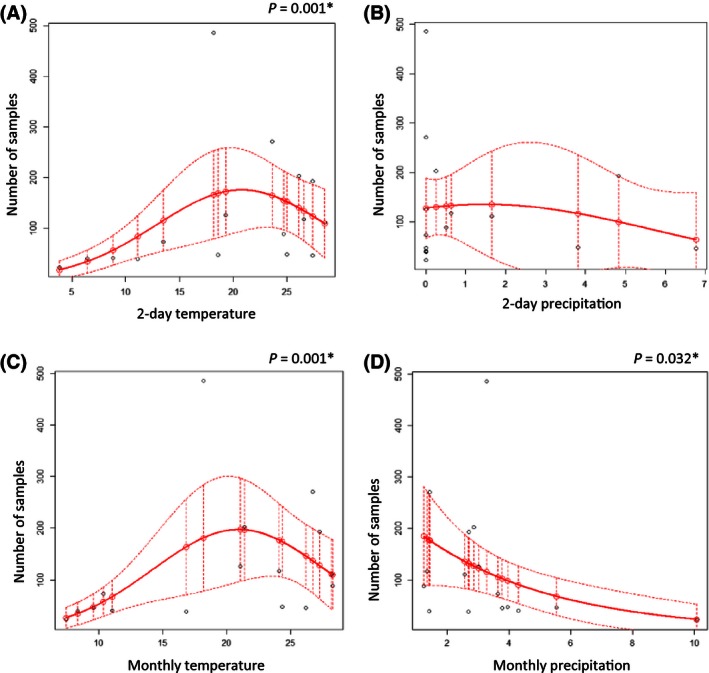
Influence of seasonal variation of climate variables on total Drosophila abundance. (A) Two‐day average temperature, (B) 2‐day precipitation average, (C) monthly average temperature, and (D) monthly average precipitation. Observed and predicted results are marked with black and red dots, respectively. Continuous line stands for a mean response, and dotted lines stand for confidence interval

Analyses of the influence of the climatic variables during the collection period, with a second‐order negative binomial GLM (sonbGLM), showed that temperature significantly influenced total number of collected samples (*p* = .001) with explained deviance (ED) of 43.9% (Fig. 4A) and a maximum abundance at a monthly average temperature of 21°C. We did not find any significant correlation between amount of precipitation during collection periods and total abundance of *Drosophila*. Combination of variables in a multivariate model did not improve the model. Taking into account monthly averages of the climatic variables, we found a significant correlation between the total number of flies and monthly average temperature (*p* = .001), using sonbGLM model that explained 44.5% of the deviance (Fig. 4C). Monthly average precipitation level significantly influenced total abundance of *Drosophila* (*p* = .032) and explained 21.1% of the deviance (Fig. 4D). In this case, the correlation is better explained by negative binomial GLM (nbGLM) then by sonbGLM. The combination of climatic variables in one multivariate model did not result in a better model.

### 3.4 Abundance individual species

#### 3.4.1 Collection period climate variables

**Figure 5:**
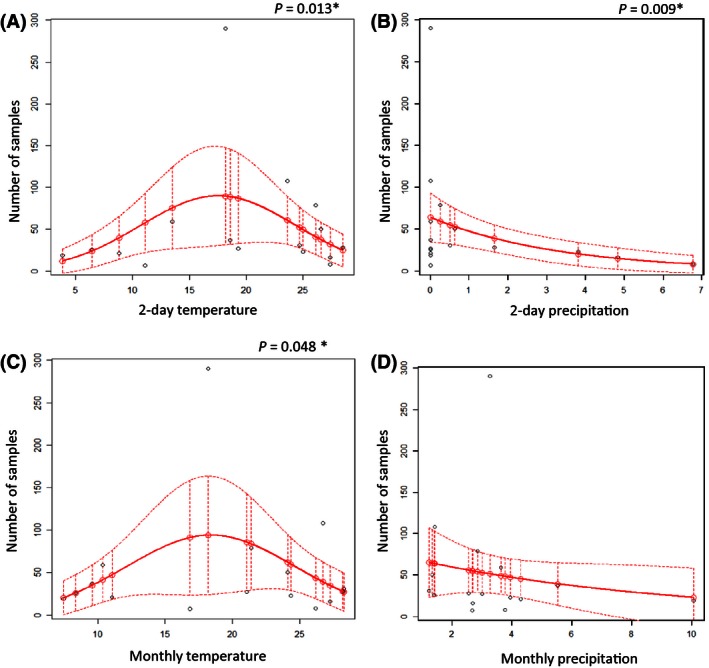
Influence of seasonal variation of climate variables on *Drosophila affinis* abundance. (A) Two‐day average temperature, (B) 2‐day precipitation average, (C) monthly average temperature, and (D) monthly average precipitation verses abundance. Observed and predicted results are marked with black and red dots, respectively. Continuous line stands for a mean response, and dotted lines stand for confidence interval

Analyzing the influence of climatic variables for the collection time period, we found a bell‐shape quadratic relation between temperature and the abundance of *D. affinis* (*p* = .01, ED = 33.5%) (Fig. 5A), *D. tripunctata* (*p* = 1.41e‐04, ED = 50.1%) (S1A), and *D. putrida* (*p* = 6.39e‐04, ED = 47.2%) (Fig. S2A). Negative binomial GLM was more efficient in explaining correlation between collection period temperature and abundance of *D. simulans* (*p* = .015, ED = 25.5%) (Fig. S3A) and *D. melanogaster* (*p* = 2.74e‐04, ED = 45.1%) (Fig. S4A). Analyzing the influence of precipitation over the collection period on the abundance of *Drosophila*, we found a significant negative correlation between precipitation and abundance of *D. affinis* (*p* = .009, ED = 28.3%, Fig. 5B). The negative binomial GLM was the most efficient model in explaining this correlation. The combination of climate variables in one multivariate model was appropriate in the case of *D. tripunctata* (*p* = 1.43e‐05, ED = 61.5%), *D. putrida* (*p* = 1.59e‐06, ED = 62.7%), and *D. affinis* (*p* = .002, ED = 42.6%).

#### 3.4.2 Monthly averages of climate variables

Considering monthly climate averages, we found that sonbGLM was the best model for explaining the bell‐shape correlation between temperature and abundance of *D. affinis* (*p* = .048, ED = 26%, Fig. 5C), *D. tripunctata* (*p* = 1.70e‐05, ED = 56%, Fig. S1C), *D. putrida* (*p* = .002, ED = 43.4%, Fig. S2C) *D. melanogaster* (*p* = 8.21e‐06, ED = 64.9%, Fig. S4C), and *D. robusta* (*p* = .002, ED = 48.2%, Fig. S5C). Influence of precipitation on *Drosophila* abundance could be best explained by the quadratic model in the cases of *D. simulans* (*p* = 2.36e‐05, ED = 58.6%, Fig. S3D), *D. melanogaster* (*p* = .04, ED = 30.2%, Fig. S4D), and *D. robusta* (*p* = 5.44e‐05, ED = 65.1%, Fig. S5D). The optimal monthly average temperature across these individual species ranged from 18 to 26°C. We found a significant positive correlation between monthly temperature average and abundance of *D. simulans* (*p* = 2.85e‐04, ED = 43.6%, Fig. S3C), as well as a negative correlation between precipitation average and abundance of *D. putrida* (*p* = .008, ED = 29%, Fig. S2D), using nbGLM. Combination of temperature and precipitation in one multivariate model resulted in a better model for *D. simulans* (*p* = 1.08e‐06, ED = 66.5%), *D. robusta* (*p* = 2.91e‐06, ED = 75%), and *D. melanogaster* (*p* = 3.45e‐06, ED = 69.9%). In addition, we did not find any significant correlation between precipitation averages during the month preceding the month of collections, with any of the models described above.

### 3.5 Influence of seasonal climatic variables on biodiversity

**Figure 6:**
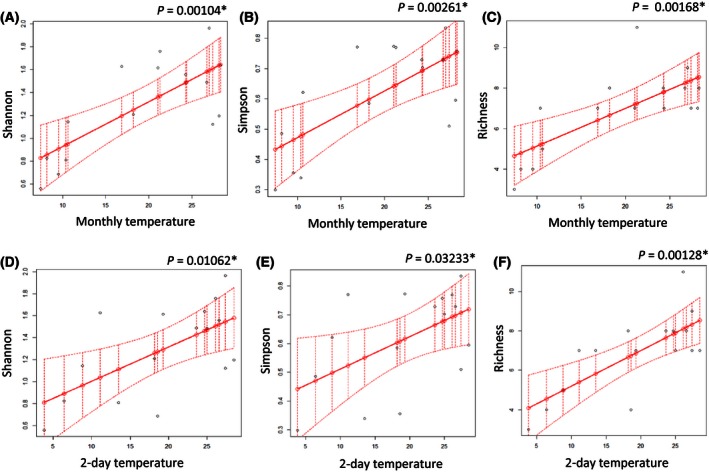
Influence of seasonal variation of climate variables on biodiversity. Monthly average temperature against: (A) Shannon index, (B) Simpson index, and (C) species richness, and 2‐day average temperature against: (D) Shannon index, (E) Simpson index, (F) species richness. Observed and predicted results are marked with black and red dots, respectively. Continuous line stands for a mean response, and dotted lines stand for confidence interval

We found a variation in the number of species collected during different seasons. Species richness was highest during the fall (September–November) with 14 species, and lowest during the winter (December–February) with only five species. During spring (March–May) and summer (June–August), we found representatives of 13 and 11 *Drosophila* species, respectively (Table 1). There was a significant positive correlation between monthly average temperature and biodiversity indices including Shannon index (*p* = .001, Fig. 6A) and Simpson index (*p* = .0026, Fig. 6B). Monthly temperature also significantly influenced species richness, with a positive correlation (*p* = .0017, Fig. 6C). Average temperature for each collection period showed a similar pattern of a positive correlation with the Shannon (*p* = .01, Fig. 6D) and Simpson (*p* = .03) biodiversity indices (Fig. 6E), as well as species richness (*p* = .007, Fig. 6F), in positive correlations. We did not find any significant correlation between amount of precipitation and *Drosophila* biodiversity.

**Table 1:** Species abundances by month

Species	June	July	August	September	October	November	December	January	February	March	April	May
*Drosophila affinis*	23	8	44	139	77	66	21	19	26	37	290	79
*Drosophila putrida*	1	3	72	69	14	2	1	0	1	2	48	34
*Drosophila tripunctata*	8	13	12	43	70	3	5	1	1	7	103	38
*Drosophila melanogaster*	7	2	13	6	7	2	0	0	0	0	0	17
*Drosophila simulans*	1	2	139	79	37	17	0	0	0	0	0	6
*Drosophila robusta*	6	5	1	0	0	2	1	0	0	0	14	16
*Drosophila suzukii*	2	5	18	12	24	13	13	3	12	1	17	0
*Zaprionus indianus*	0	0	0	8	12	5	0	0	0	0	0	0
*Drosophila busckii*	0	0	0	0	0	0	0	0	0	0	0	2
*Drosophila cardini*	0	0	0	0	1	0	0	0	0	0	0	0
*Drosophila euronotus*	0	1	0	0	1	1	0	0	0	0	0	2
*Drosophila falleni*	0	0	0	0	0	0	0	0	0	0	5	1
*Drosophila immigrans*	0	0	0	0	0	0	0	0	0	0	6	6
*Drosophila macrospina*	0	0	3	0	0	1	0	0	0	0	2	0
*Drosophila nigromelanica*	0	7	1	2	0	0	0	0	0	0	0	2
*Hirtodrosophila duncani*	0	0	0	1	0	0	0	0	0	0	0	0
*Mycodrosophila dimidiata*	0	0	1	0	0	0	0	0	0	0	0	0
*Scaptomyza frustulifera*	0	0	0	1	0	0	0	0	0	0	0	0

### 3.6 Influence of land use on abundance and biodiversity of *Drosophila*

**Figure 7:**
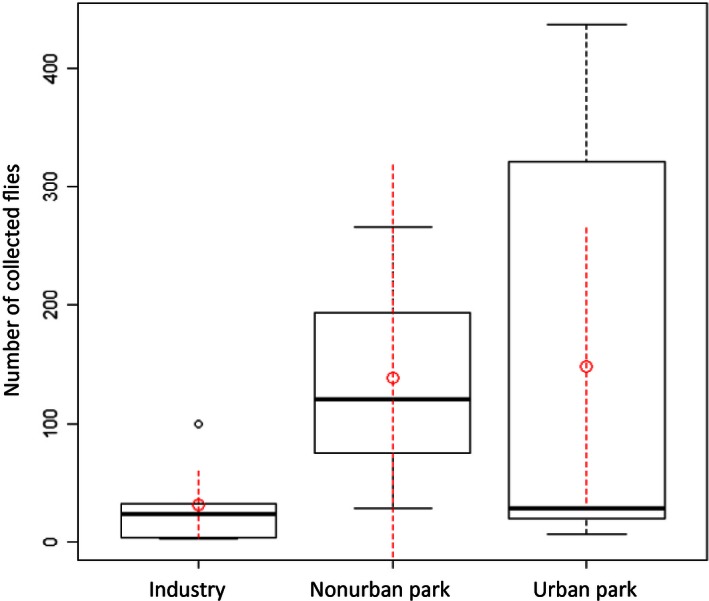
Number of individual flies collected per land‐use category. The lowest number of samples was collected in industrial zones. The highest number of samples was collected in urban park areas

Separating our sites into three categories, industrial areas, urban parks, and nonurban parks, we found a significant correlation between land use and total abundance of *Drosophila* species (*p* = .037, Fig. 7). The analyses of most represented species individually showed a correlation between abundance of *D. tripunctata* (*p* = .008) and land‐use categories. In addition, land‐use type may influence abundance of *D. putrida* (*p* = .079). The lowest abundance of both of these mushroom‐feeding *Drosophila* was recorded from industrial areas. Disturbance score, which evaluated the presence or absence of disturbance factors such as garbage or trash cans, industrial or agricultural productions, asphalt roads, highway, railroads or airports, and residential or public catering buildings across all collection sites, only correlated with the abundance of *D. putrida* (*p* = .035), where greater disturbance produced lower abundances of the fly species. In contrast to abundance, we did not find any significant correlation between land use and biodiversity indices or species richness. In addition, we did not find any evidence to conclude that narrow range of latitude or longitude over the collection sites produced a significant effect on abundance or biodiversity of *Drosophila*.

## 4 Discussion

The previous *Drosophila* biodiversity studies in Alabama reported 26 Drosophilidae species (Sturtevant, 1916, 1918; Sturtevant, 1921) compared to the 18 found in this study, a difference in species richness of only five species. This difference could be due to the sampling techniques or/and sampling area choice. In previous biodiversity surveys, Sturtevant (1916, 1918), sampling was mostly performed via catching samples in a banana bait and net sweeping of fruits and mushrooms. Sturtevant and Dobzhansky (1936) noticed that at least for *D. affinis* subgroup species, they did not observe a significant difference in species frequencies based on the collection method. In addition, Dobzhansky and Pavan (1950) indicated that North American mycophagous *Drosophila* readily came to banana baits and we also found that the mycophagous flies were found on our banana baits in our study.

Sturtevant performed the Alabama collections cited in his 1918 paper throughout the year (April, June, July, October, November) between 1914 and 1916, thus covering a similar seasonal range to our own study. The Sturtevant (1918) study aimed to describe the diversity of *Drosophila* and other dipterous insects collected in Alabama, and the study was conducted in and around Mobile, Alabama. Mobile County and Tuscaloosa County are approximately 195 miles apart, and given the broad distribution of most of these *Drosophila* species (Markow & O'Grady, 2005), it is likely that many of the species should be found at both sites if they are present at all; however, further work would be needed to clarify how spatial variation influenced local species diversity changes in Mobile County specifically. Based on these observations, we believe that our sampling techniques, which were broadly similar to earlier studies, should not substantially influence the species composition difference that we have noticed between previous and current survey. However, it is still possible the differences in species presence between what was observed 100 years ago and now could be due, in part, to random variation in collecting success, while also being influenced by more deterministic factors such as climate change and urbanization of the available habitats.

We saw a significant change in species composition. Almost half of the species that we identified in this study were not reported during the last biodiversity survey: *H. duncani*,* S. frustfrustulifera*,* L. angusta*,* Z. indianus*,* D. macrospina*,* D. nigromelanica*,* D. suzukii*,* D. euronotus*,* D. cardini*, and *D. falleni*. However, before 1960 in North America, *D. falleni* was mistakenly lumped with *D. transversa* (which Sturtevant, 1918 reported finding in Alabama), and thus *D. falleni* might actually be a native species to Alabama and part of Sturtevant's original collections (Wheeler, 1960). Of ten previously unreported species, *H. duncani*,* S. frustfrustulifera*, and *D. cardini* were captured only once, which makes it difficult to ascertain their permanent presence in Alabama. Overall, it is reasonable to conclude that there has been a substantial change in Drosophilidae species diversity in Alabama over the last century.

### 4.1 Invasive species

Among the newly introduced *Drosophilas*, the most interesting was the presence of *D. suzukii* and *Z. indianus* in our collected samples. Both of these flies are recognized as invasive pests, and both were consistently present in our traps. We were able to find *D. suzukii* during 13 of 16 collection trips and *Z. indianus* in four collection trips from September to November. *Drosophila suzukii* was officially reported in Alabama in 2012 (Burrack et al., 2012), but there had not been a published report of *Z. indianus* presence in Alabama. *Zaprionus indianus* was first reported as a pest species in 1999 in Brazil, where it destroyed 40% of commercial fig production in the state of São Paulo (Commar et al., 2012). In the United States, *Z. indianus* was reported in Florida in 2006 (van der Linde et al., 2006) and Pennsylvania in 2014 (Joshi et al., 2014). It also was reported in Mexico (Lasa & Tadeo, 2015) and Canada (Renkema, Miller, Fraser, Légaré, & Hallett, 2013). Within the United States, *Z. indianus* was found on peach, raspberry, and blueberry farms (Biddinger, Joshi, & Demchak, 2012; Joshi et al., 2014). In Alabama, peach and blueberry production is not very substantial and valued at <4 million US dollars in 2014 (USDA). In addition, *Z. indianus* usually infests only damaged fruits and is considered a secondary pest (Joshi et al., 2014). Due to these reasons, *Z. indianus* would not be expected to become a major pest in Alabama. However, one possible concern could be an overlap of the host range of *D. suzukii* and *Z. indianus* in Alabama, which could allow *Z. indianus* to colonize fruits damaged by *D. suzukii*.

### 4.2 Native species

Among native species, we could see some pattern between *Drosophila* seasonal abundance and their host dietary types. *Drosophila affinis* was most abundant across all months with the exception of August. *Drosophila affinis* is a generalist species that feeds on tree saps, fruits, and mushrooms (Carson & Stalker, 1951; Strickberger, 1962; Sturtevant, 1916) and prefers fruits and slime fluxes for oviposition sites (Avondet, Blair, Berg, & Ebbert, 2003). *Drosophila affinis* has a rather wide distribution range within the United States, with sightings as far north as Maine and Quebec (Jaenike, 1978; Miller, 1958). The presence of at least one food type during all the seasons, a broad range of oviposition hosts, and the relatively high cold tolerance of *D. affinis* could at least partially explain its highest abundance during most of the year.

*Drosophila tripunctata* and *D. putrida* are primarily fungus feeders that choose mushrooms as their preferred breeding sites (Sturtevant, 1916; Strickberger, 1962; Avondet, Blair, Berg, & Ebbert, 2003). *Drosophila tripunctata* is less discriminate in food preference and can be found on rotten fruits and slime fluxes (Carson & Stalker, 1951). During our collections, both of these species were abundant in banana traps, suggesting that *D. putrida* could use rotten fruits as a food source in natural environments. Both of these species were found during nearly all collection months. Together, these generalist mycophagous species would make the second most abundant group of flies across our samples, which suggests that their diverse host range could be responsible for high abundance during all seasons.

Three relatively low abundance species were *D. simulans*,* D. melanogaster,* and *D. robusta,* and they all have a relatively narrow host range. *Drosophila simulans* and *D. melanogaster* primarily feed on rotten fruits (Strickberger, 1962; Sturtevant, 1916), and rotten fruit is also the preferred oviposition media for these species (Avondet, Blair, Berg, & Ebbert, 2003; Carson & Stalker, 1951). *Drosophila simulans* and *D. melanogaster* were absent from our traps from December to April, which could be correlated with fruit and berry season. *Drosophila robusta* feeds primarily on fruits, mushrooms, and tree saps (Carson & Stalker, 1951; Strickberger, 1962; Sturtevant, 1916), is very specific in choosing sites for oviposition, and in natural environments was reported to breed primarily on slime fluxes (Carson & Stalker, 1951; Avondet, Blair, Berg, & Ebbert, 2003). Abundance of *D. robusta* did not follow any obvious seasonal pattern, and the species was absent from our traps in September and October, and from January to March.

Based on our observations overall, we can conclude that the ability to use a broad host range for feeding and oviposition could play an important role in abundance of *Drosophila* species through all seasons in Alabama.

### 4.3 Climatic impacts on abundance and biodiversity

According to our models, monthly temperature influences abundance more than the 2‐day collection period temperature for total abundance of *Drosophila,* as well as abundance of *D. tripunctata*,* D. melanogaster*,* D. robusta*, and *D. simulans*. The effect of monthly temperature on total abundance was driven largely by the highly abundant *D. affinis*. The average amount of precipitation per month also influences total abundance and that of the most represented species of *Drosophila*, with the exception of *D. affinis*. The lack of a significant monthly precipitation effect on the *D. affinis* that we collected is especially interesting as *D. affinis* consisted of a substantial proportion of all collections, meaning that the other species were driving the variation in total abundance in response to monthly. We can conclude that difference in climate variables per month produces a more significant effect on the abundance of *Drosophila* than temperature and precipitation during collection days. In addition, we can see that most *Drosophila* species exhibit a quadratic response to a seasonal climate variation, suggesting the presence of an optimal climatic condition range for each species.

It was shown that precipitation had a positive correlation with mushroom's productivity, which might result in increased abundance of mycophagous flies (Krebs, Carrier, Boutin, Boonstra, & Hofer, 2008; Worthen & McGuire, 1990). Therefore, assuming that increased precipitation levels would facilitate fungi's fruiting body formation, we tested the influence of precipitation during the 56‐ to 28‐day period preceding the collection on the abundance of mycophagous *D. tripunctata* and *D. putrida*. However, we did not find any significant correlation. The lack of correlation can probably be explained by combination of factors in mushroom and *Drosophila* ecology. Worthen and McGuire (1990) observed that rainfall could produce a significant effect on the following week's mushroom abundance and noticed that an individual fungi's fruit body is often short‐lived. At 18°C, *D. putrida* and *D. tripunctata* egg to adult developmental time ranges from 14 to 15 days and might be shortened by warmer temperatures (Markow & O'Grady, 2005); thus, we might expect a spike in abundance to occur within 2–3 weeks of the increase in mushroom fruit bodies. Boulétreau (1978) described that almost half of female *Drosophila melanogaster* collected from natural populations were <24–36 hr old, while Roff (1980) suggested that in wild, adult *Drosophila* life span might be only few days; thus, any spike in abundance due to an increase in mushroom fruiting bodies might be expected to be of a short duration, on the order of a few days.

Based on the limited information about wild *Drosophila* life span and rate of mushrooms’ fruit body productivity increase in the response to rainfall, it is logical that monthly precipitation during the collection month would produce more effect on mycophagous *Drosophila* populations than precipitation in the previous month. However, surprisingly, the abundance of the two mycophagous species was actually negatively correlated with precipitation within the collection month (statistically significant for *D. putrida*). The lack of a clear association between host availability and mycophagous fly abundance at the monthly scale suggests that more granular analyses are needed in future studies to determine how and whether the fly abundance is influenced by the presence of mushrooms.

Temperature influences not only mature *Drosophila* activity but also its developmental time and larval survival (Crill, Huey, & Gilchrist, 1996; James, Azevedo, & Partridge, 1997). If temperature fluctuations during the month are out of a species’ optimal range, then fewer mature *Drosophila* will develop. This could generally explain the greater influence of monthly temperature on abundance of *Drosophila*. Interestingly, we did not find a significant correlation between abundance of *D. suzukii* or *Z. indianus* and any of the climate variables. In the case of *Z. indianus,* the major reason for this lack of correlation could be small sample size (25 flies) and our ability to find this pest fly only during fall months. However, *D. suzukii* was present most times of the year, which suggests that this pest species has physiological or behavioral adaptations to better resist differences in seasonal climate change than native *Drosophila* species.

Several previous studies reported no significant correlation between abundance of *Drosophila* and seasonal temperature variation (Guruprasad et al., 2010; Srinath & Shivanna, 2014; Torres & Madi‐Ravazzi, 2006). The reason for the difference in our results relative to theirs could be due, in part, to the different approach in statistical analyses. Most of these studies used a linear regression model. However, living organisms have an optimal range of climate conditions for their survival and reproduction that is not linear (Kindt & Coe, 2005), and different species of *Drosophila* exhibit different temperature tolerance (Goto & Kimura, 1998; Hoffmann, 2010; Kellermann et al., 2012). In addition, Poppe, Valente, & Schmitz, (2013) showed negative correlation between *Drosophila* abundance and maximum/minimum temperatures, which further suggests that *Drosophila* are mostly abundant in a temperature range between the extreme values. The quadratic model appears to be the most appropriate for analyzing the influence of temperature on the abundance of *Drosophila* species and allowed us to identify optimal condition ranges. For the most abundant species, their optimal monthly average temperature ranged from 18 to 26°C. In addition, ecological data are often over‐dispersed (Kindt & Coe, 2005), and several studies indicate that a negative binomial model, as we used in this study, and the quasi‐Poison model are more appropriate in analyses of such data (O'Hara & Kotze, 2010; Ver Hoef & Boveng, 2008).

Other possible sources of difference between our study and the past test of ecological effects on *Drosophila* species are the different climate zones in which the studies were carried out. Alabama exhibits subtropical climate, and the temperature range during our study was approximately 25°C. In tropical regions, the temperature range could be smaller and would not produce such significant effects (Dobzhansky & Pavan, 1950; da Mata et al., 2015). In addition, several studies took into account only temperature and precipitation levels measured during collection periods (Poppe, Valente, & Schmitz, 2013; Srinath & Shivanna, 2014; Torres & Madi‐Ravazzi, 2006). In this study, we indicated that changes in average monthly environmental variables can influence abundance of *Drosophila* in a more significant way than changes during the collection period. In addition, our survey found different species composition than found in other studies, and their inherent species‐specific biology could be influenced by ecological conditions in distinct ways (Hoffmann, 2010; Kellermann et al., 2012).

### 4.4 Land‐use impacts on abundance and biodiversity

We were able to find significant correlation between land use and abundance of *Drosophila* species. The highest number of samples per trap came from urban park areas and could be explained by abundance of food sources for generalist *Drosophila* as a result of human refuse and a relatively high amount of vegetation that could provide a shelter (Ferreira & Tidon, 2005; van Klinken & Walter, 2001). The strongest correlation between land use and individual species abundance was shown by species that tend to use mushrooms as food and breeding substrate: *D. putrida* and *D. tripunctata*. In urban areas, biodiversity of fungal communities is lower than in rural areas (Egerton‐Warburton & Allen, 2000; Newbound, Mccarthy, & Lebel, 2010), which potentially can influence abundance of mycophagous *Drosophila*.

**Figure 8:**
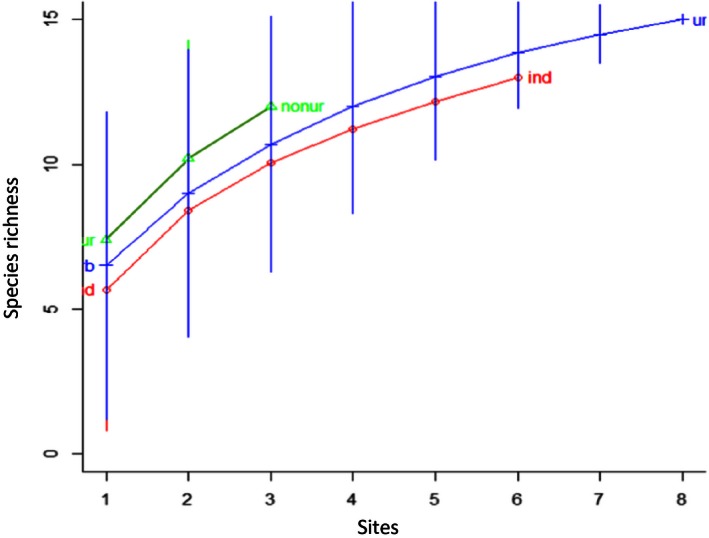
Species accumulation curves. Made with random accumulation method via 10,000 permutations for land‐use categories: Circles represent industrial areas, triangles represent nonurban parks, and crosses represent urban parks

In contrast to abundance measures, we found no significant correlation between *Drosophila* biodiversity and land use. A species accumulation curve that was made via a random accumulation method suggested that if we take into account only three sites per category (which is the maximum number of sites for nonurban parks), then the most natural environment should have had the highest number of species (Fig. 8). This is consistent with the idea that in a more undisturbed environment the number of rare *Drosophila* species would be higher (Parsons, 1991). Unfortunately, only the urban park zones were sampled at a sufficient number of sites to saturate species detection according to rarefaction analysis, which plots the number of species as a function of the sample's number and allows evaluation of the sufficiency of sample size (Kindt & Coe, 2005, Fig. S6).

## 5 Conclusion

Global climate change can cause a shift in the composition and functioning of biological communities (Cardinale et al., 2006; Parsons, 1991; Root et al., 2003), and as exotic species extend their habitat range they often replace endemic organisms (Ferreira & Tidon, 2005; Hooper et al., 2005). In this study, we analyzed biodiversity and species abundance of *Drosophila* in Central Alabama. Nearly half of the identified species we found were not reported during the last major biodiversity surveys (Sturtevant, 1918, 1921), which suggests a change in Alabama's *Drosophila* species composition overall the last 100 years. It has been shown that endemic *Drosophila* species are usually sensitive to climatic variable changes (Parsons, 1991), and we found a significant correlation between the most abundant endemic *Drosophila* species and seasonal shifts in temperature and precipitation. We also found that biodiversity overall was influenced by seasonal temperature variation. Surprisingly, analyses of invasive pest species (*Z. indianus* and *D. suzukii*) did not show any significant correlation between their abundance and seasonal climate variables, suggesting that other factors, such as human influence, drive their abundances.

Urbanization often leads to destruction of natural habitats and significant changes in biodiversity and abundance of endemic and specialist species (Ferreira & Tidon, 2005; Parsons, 1991), and in our study, we found a significant correlation between land‐use type and abundance of *Drosophila*. In addition, the majority of collected flies were representatives of generalist species. Our results suggest a significant change in *Drosophila* species composition and the absence of many historically endemic species in the subtropical region of Alabama. To better survey the whole biodiversity of *Drosophila* in Alabama, more collections should be performed across the state. Given the growing level of urbanization, we expect that cosmopolitan species of *Drosophila* such as *D. simulans*,* D. suzukii*, and especially *Z. indianus* will become more abundant in Alabama and could establish themselves as dominant species in urban environments.

## Funding Information

National Institutes of Health (Grant/Award Number: “National Institutes of Health: 5R01GMO98856”).

## Conflict of Interest

None declared.
