# Supplementary material for: The changing biodiversity of Alabama Drosophila: important impacts of seasonal variation, urbanization, and invasive species
Source: Ecol Evol. 2016 Sep 12;6(19):7057–69. doi: 10.1002/ece3.2452 (PMC5120374; doi:10.1002/ece3.2452)

| Collection sites | Latitude | Longitude | *D. affinis* | *D. robusta* | *D. melanogaster* | *D. simulans* | *D. tripunctata* | *D. putrida* | *D. suzukii* | *Z. indianus* | *D. busckii* | *D. cardini* | *D. euronotus* | *D. falleni* | *D. immigrans* | *D. macrospina* | *D. nigromelanica* | *H. duncani* | *M. dimidiata* | *S. frustulifera* |
| --- | --- | --- | --- | --- | --- | --- | --- | --- | --- | --- | --- | --- | --- | --- | --- | --- | --- | --- | --- | --- |
| Tuscaloosa airport | 33.224863 | -87.601892 | 1 | 0 | 1 | 0 | 0 | 0 | 0 | 0 | 0 | 0 | 0 | 0 | 0 | 0 | 0 | 1 | 0 | 0 |
| Apartament complex | 33.16152 | -87.501599 | 3 | 0 | 0 | 0 | 7 | 8 | 3 | 0 | 0 | 0 | 0 | 0 | 0 | 0 | 0 | 0 | 0 | 0 |
| Arboretum | 33.191524 | -87.474854 | 43 | 8 | 2 | 5 | 28 | 26 | 1 | 1 | 0 | 0 | 0 | 1 | 3 | 0 | 3 | 0 | 0 | 0 |
| Archaeological park | 33.002734 | -87.628048 | 7 | 0 | 0 | 0 | 5 | 0 | 3 | 0 | 0 | 0 | 0 | 0 | 0 | 0 | 0 | 0 | 0 | 0 |
| Capitol park | 33.185117 | -87.54568 | 165 | 21 | 5 | 77 | 89 | 30 | 35 | 0 | 0 | 0 | 1 | 5 | 2 | 0 | 0 | 0 | 0 | 0 |
| JVC plant | 33.173133 | -87.459782 | 1 | 0 | 0 | 2 | 0 | 1 | 0 | 0 | 0 | 0 | 0 | 0 | 0 | 0 | 0 | 0 | 0 | 0 |
| Kentuck park | 33.219441 | -87.590388 | 3 | 0 | 0 | 2 | 6 | 7 | 8 | 0 | 0 | 0 | 0 | 0 | 0 | 0 | 0 | 0 | 0 | 0 |
| Lake lurleen | 33.301699 | -87.670348 | 9 | 0 | 0 | 1 | 3 | 6 | 2 | 0 | 0 | 0 | 0 | 0 | 0 | 0 | 8 | 0 | 0 | 0 |
| Mercedez plant | 33.183258 | -87.257463 | 18 | 0 | 1 | 3 | 2 | 5 | 3 | 0 | 0 | 0 | 0 | 0 | 0 | 0 | 1 | 0 | 0 | 0 |
| Monish park | 33.198731 | -87.54312 | 11 | 0 | 0 | 8 | 0 | 1 | 0 | 5 | 0 | 0 | 0 | 0 | 0 | 0 | 0 | 0 | 0 | 0 |
| Old Park | 32.215 | -87.5656 | 4 | 0 | 0 | 0 | 1 | 1 | 0 | 0 | 0 | 0 | 0 | 0 | 0 | 1 | 0 | 0 | 0 | 0 |
| Farm | 32.8825 | -87.6803 | 14 | 0 | 1 | 3 | 8 | 8 | 1 | 0 | 0 | 0 | 0 | 0 | 0 | 0 | 0 | 0 | 0 | 0 |
| Rest area | 33.1883 | -87.3258 | 6 | 0 | 1 | 2 | 0 | 11 | 2 | 0 | 0 | 0 | 0 | 0 | 0 | 0 | 0 | 0 | 0 | 0 |
| Park at Manderson landing | 33.21841 | -87.554695 | 67 | 0 | 4 | 98 | 12 | 18 | 2 | 9 | 0 | 0 | 0 | 0 | 0 | 0 | 0 | 0 | 0 | 1 |
| Shelby park | 33.215175 | -87.541851 | 5 | 0 | 0 | 0 | 24 | 1 | 0 | 2 | 0 | 0 | 0 | 0 | 0 | 0 | 0 | 0 | 0 | 0 |
| Nucor Steel | 33.241044 | -87.503232 | 58 | 7 | 2 | 4 | 10 | 11 | 3 | 3 | 0 | 0 | 1 | 0 | 1 | 0 | 0 | 0 | 0 | 0 |
| Tanglewood | 32.8545 | -87.6706 | 169 | 3 | 0 | 8 | 10 | 70 | 5 | 0 | 0 | 0 | 1 | 0 | 0 | 0 | 0 | 0 | 0 | 0 |
| Tire plant | 33.192501 | -87.605556 | 4 | 1 | 1 | 11 | 0 | 0 | 6 | 0 | 0 | 0 | 0 | 0 | 0 | 0 | 0 | 0 | 0 | 0 |
| Industrial products storage | 33.2001 | -87.5736 | 3 | 0 | 1 | 0 | 0 | 0 | 0 | 4 | 0 | 0 | 1 | 0 | 0 | 0 | 0 | 0 | 0 | 0 |
| Road side | 33.226111 | -87.540833 | 11 | 0 | 5 | 29 | 5 | 5 | 8 | 0 | 0 | 0 | 0 | 0 | 0 | 0 | 0 | 0 | 0 | 0 |
| University of Alabama | 33.211901 | -87.543881 | 219 | 5 | 26 | 25 | 80 | 33 | 36 | 0 | 0 | 0 | 1 | 0 | 6 | 5 | 0 | 0 | 1 | 0 |
| Veteran park | 33.203884 | -87.524929 | 1 | 0 | 4 | 3 | 3 | 3 | 0 | 0 | 0 | 1 | 0 | 0 | 0 | 0 | 0 | 0 | 0 | 0 |
| Phifer Incorporated | 33.167862 | -87.563923 | 7 | 0 | 0 | 0 | 11 | 2 | 2 | 1 | 2 | 0 | 0 | 0 | 0 | 0 | 0 | 0 | 0 | 0 |

Table S1: Number of samples per species for each collection location (with recorded longitude and longitude).


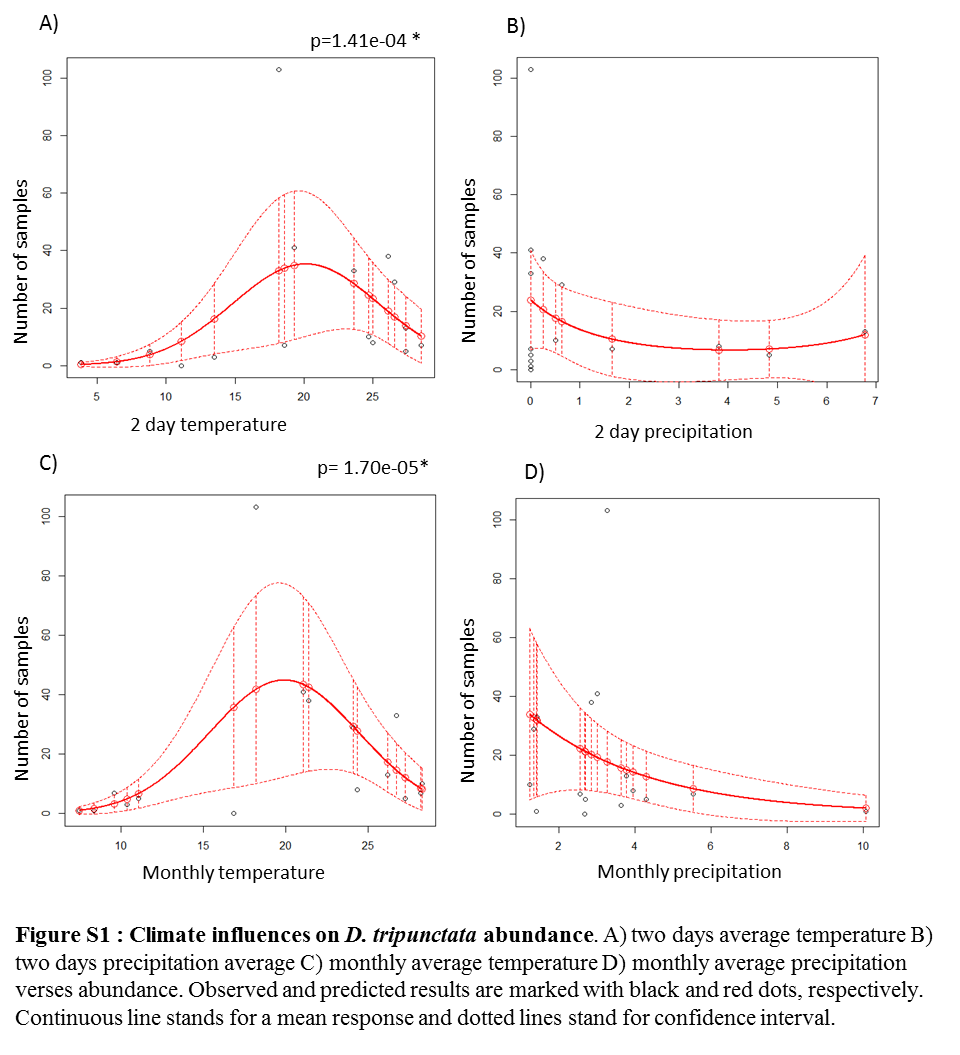


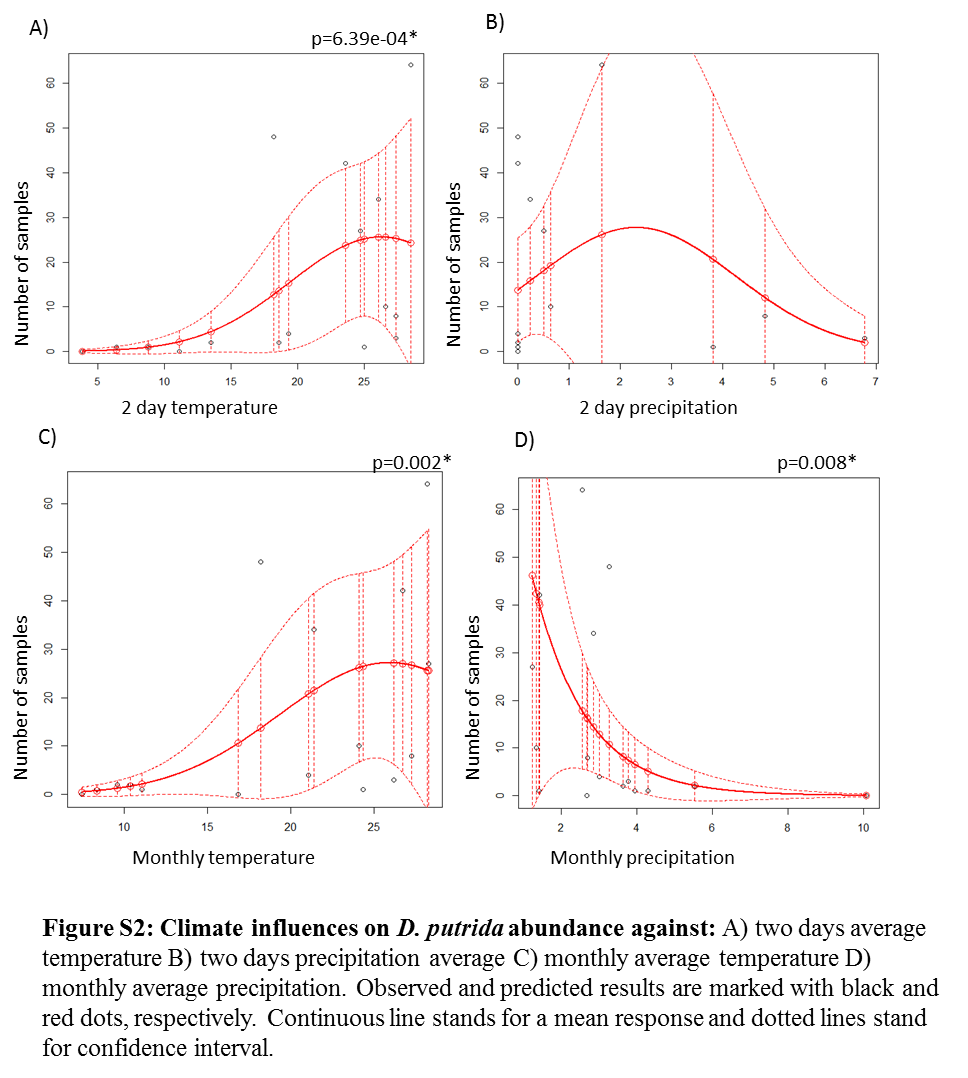


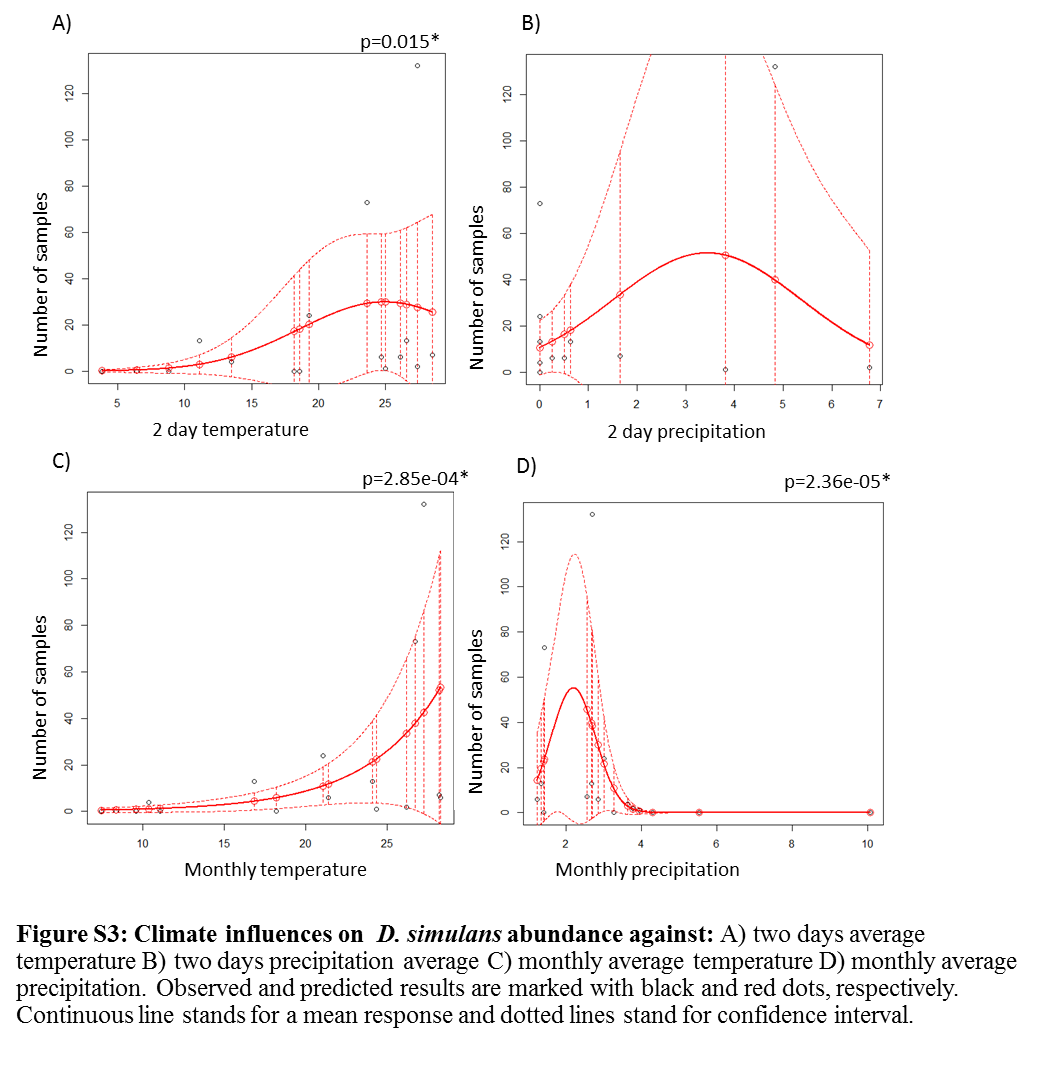


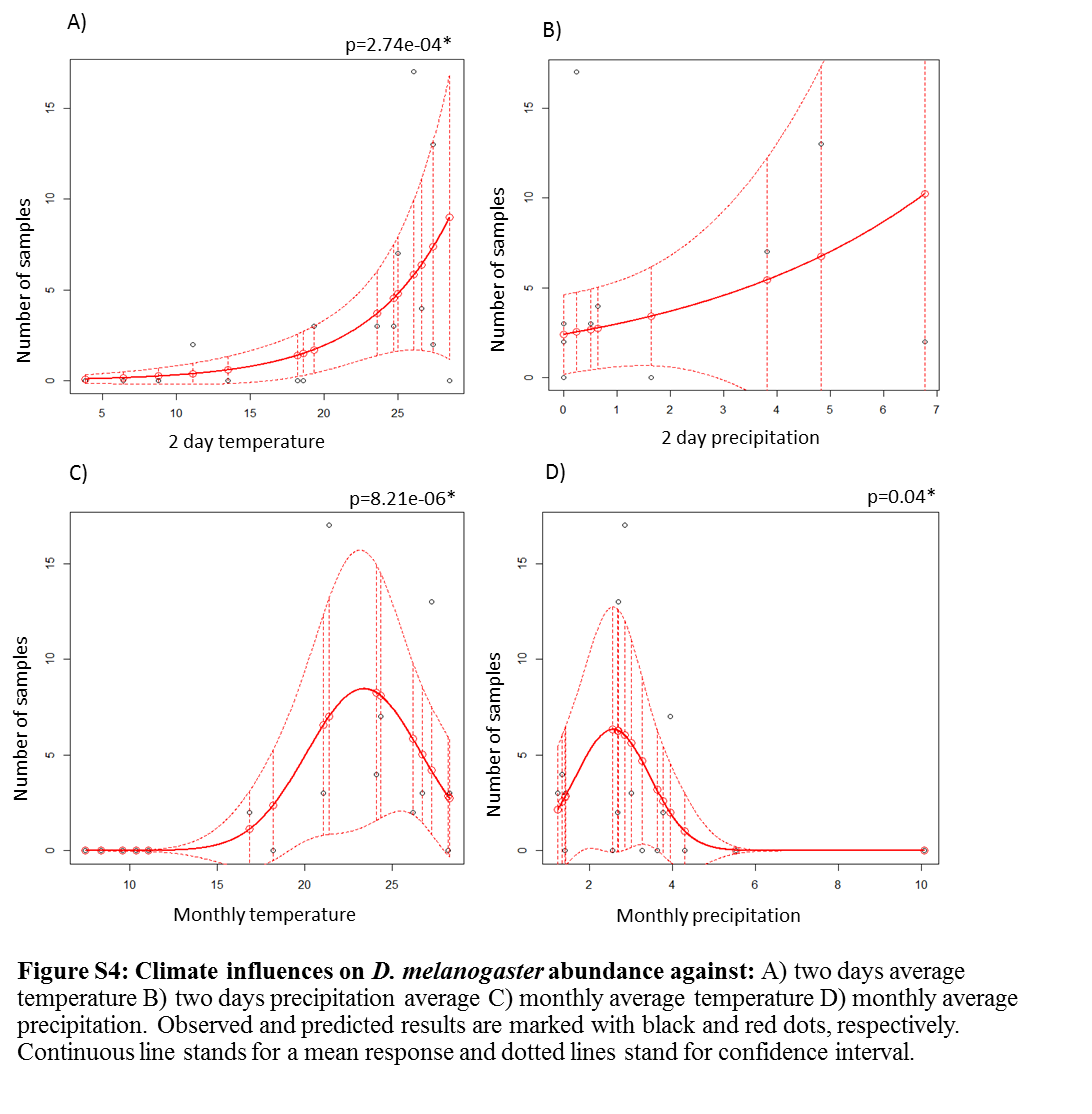


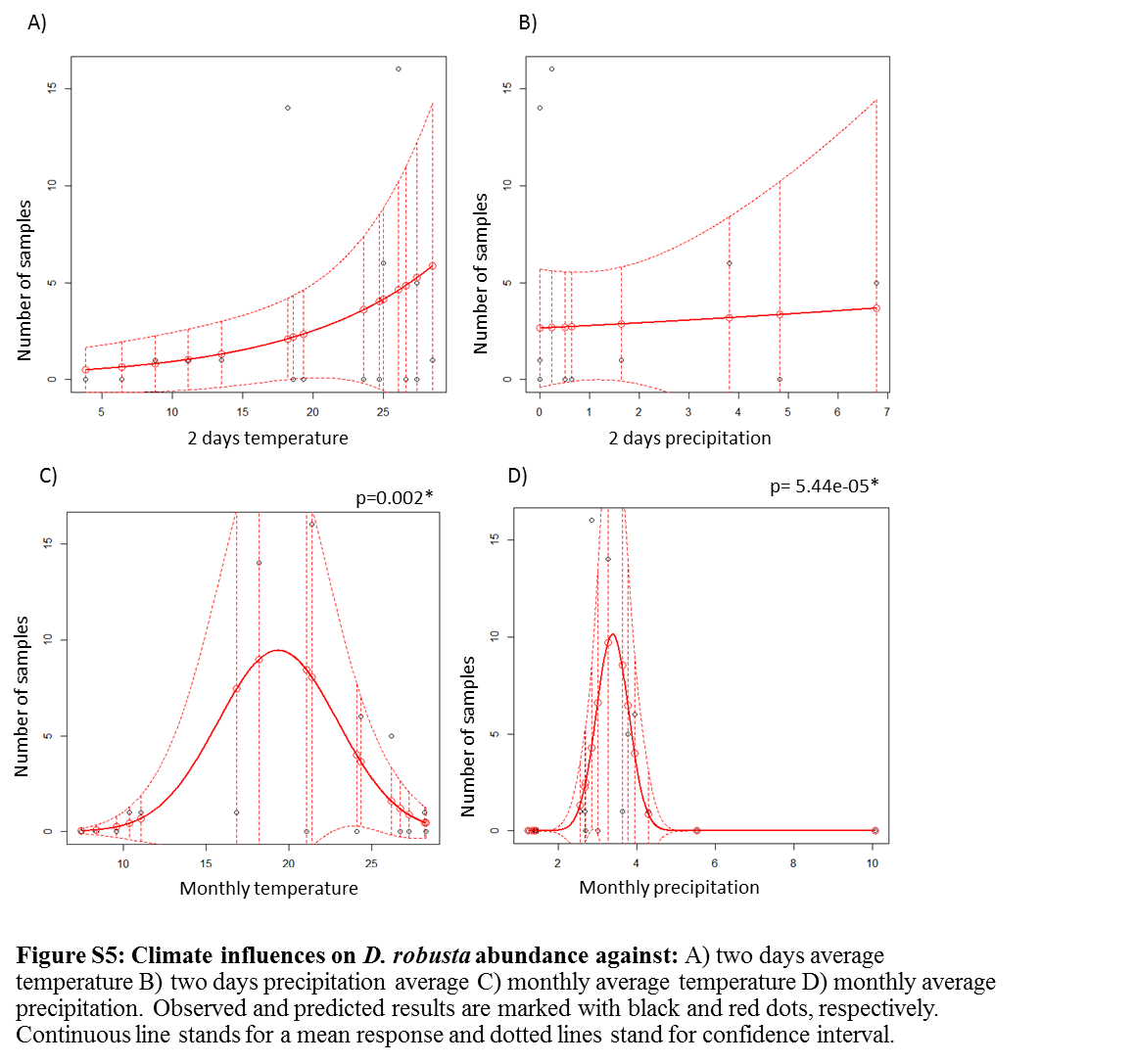


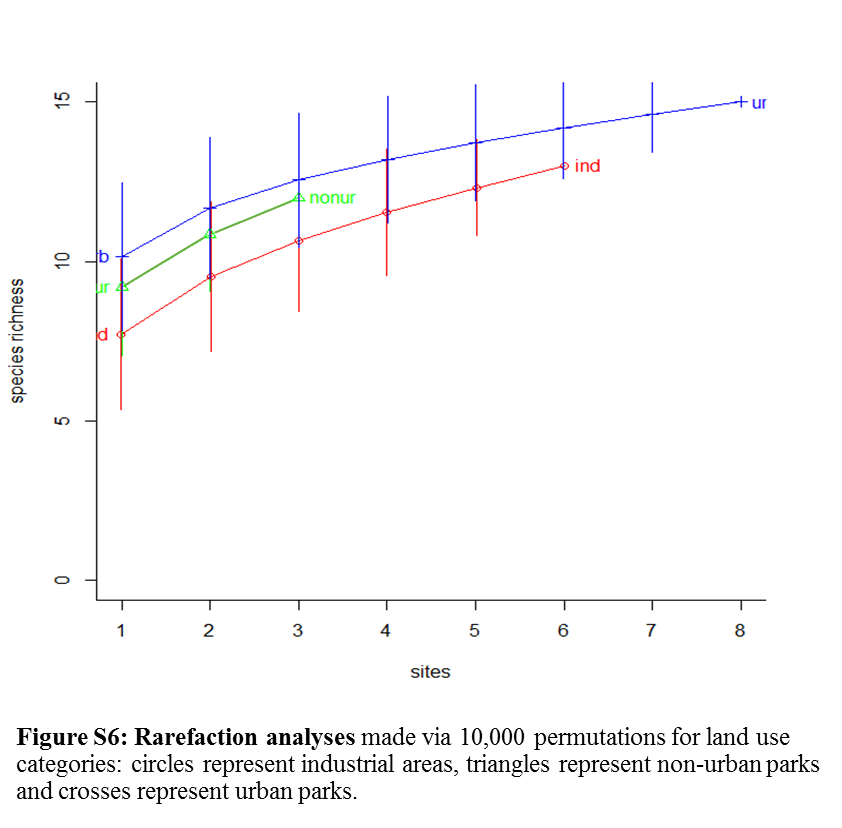

Supplement: Supplementary file 1 [file ECE3-6-7057-s001.docx]
